# GENETAG: a tagged corpus for gene/protein named entity recognition

**DOI:** 10.1186/1471-2105-6-S1-S3

**Published:** 2005-05-24

**Authors:** Lorraine Tanabe, Natalie Xie, Lynne H Thom, Wayne Matten, W John Wilbur

**Affiliations:** 1 National Center for Biotechnology Information, National Library of Medicine, NIH, 8600 Rockville Pike, Bethesda, MD 20894, USA; 2 Consolidated Safety Services, 10335 Democracy Lane, Suite 202, Fairfax, VA 22030, USA

## Abstract

**Background:**

Named entity recognition (NER) is an important first step for text mining the biomedical literature. Evaluating the performance of biomedical NER systems is impossible without a standardized test corpus. The annotation of such a corpus for gene/protein name NER is a difficult process due to the complexity of gene/protein names. We describe the construction and annotation of GENETAG, a corpus of 20K MEDLINE^® ^sentences for gene/protein NER. 15K GENETAG sentences were used for the BioCreAtIvE Task 1A Competition.

**Results:**

To ensure heterogeneity of the corpus, MEDLINE sentences were first scored for term similarity to documents with known gene names, and 10K high- and 10K low-scoring sentences were chosen at random. The original 20K sentences were run through a gene/protein name tagger, and the results were modified manually to reflect a wide definition of gene/protein names subject to a specificity constraint, a rule that required the tagged entities to refer to specific entities. Each sentence in GENETAG was annotated with acceptable alternatives to the gene/protein names it contained, allowing for partial matching with semantic constraints. Semantic constraints are rules requiring the tagged entity to contain its true meaning in the sentence context. Application of these constraints results in a more meaningful measure of the performance of an NER system than unrestricted partial matching.

**Conclusion:**

The annotation of GENETAG required intricate manual judgments by annotators which hindered tagging consistency. The data were pre-segmented into words, to provide indices supporting comparison of system responses to the "gold standard". However, character-based indices would have been more robust than word-based indices. GENETAG Train, Test and Round1 data and ancillary programs are freely available at .  A newer version of GENETAG-05, will be released later this year.

## Background

The automatic identification of gene and protein names in the MEDLINE^® ^database of literature citations is a challenging named entity recognition (NER) task. Biomedical NER has been an active research area for some time. Systems capable of high performance on this task are desirable because NER precedes other tasks including information extraction and text mining. The apparent simplicity of the gene/protein NER task conceals its inherent complexity stemming from an often unconventional and ambiguous genetic nomenclature.

We have previously developed AbGene, a gene/protein name tagger trained on MEDLINE abstracts using a combination of statistical and rule-based strategies. Due to the difficulty of manually evaluating AbGene results, we needed to create a tagged corpus for evaluating the performance of AbGene applied to full text articles. The GENIA corpus version 3.0 contains a total of 93,293 biological terms annotated by two domain experts [1]. However, it was not suitable for our purposes because we ran AbGene on unrestricted text, and the GENIA corpus is restricted to text retrieved using the search terms *human*, *blood cell *and *transcription factor*. Additionally, the entities in GENIA are allowed to be generic, whereas AbGene was designed to extract specific gene/protein names only.

One fundamental problem in corpus annotation is the definition of what constitutes an entity to be tagged. For example, the MUC-7 Named Entity Task to identify organizations, persons and locations in text necessitated the lengthy MUC-7 Named Entity Task Definition, which specify the rules for annotating each entity [2]. The following excerpts from the MUC-7 Named Entity Task Definition exemplify the complexity of the annotation process:

### A.1.1 Entity-expressions that modify non-entities

Entity names used as modifiers in complex NPs that are not proper names are to be tagged when it is clear to the annotator from context or the annotator's knowledge of the world that the name is that of an organization, person, or location.

### A.1.3 Entity-strings embedded in entity-Expressions

In some cases, multi-word strings that are proper names will contain entity name substrings; such strings are not decomposable; therefore, the substrings are not to be tagged. (See A.1.2 re special cases involving prenominal modifiers of person identifiers.)

### A.1.6.2 The definite article in an alias

When a definite article is commonly associated with an alias, it also must be tagged.

<ENAMEX TYPE="PERSON">The Godfather</ENAMEX>

However, the scoring program ignores a certain list of premodifiers as specified in section 3.3 which may make the scoring in some of these cases more lenient than this rule implies. The scorer does *not* ignore those premodifiers within quotation marks such as inside the tags in A.1.6.1 above.

The developers of the GENIA corpus followed a less exacting annotation strategy that did not allow determiners, ordinals nor cardinals to appear in tagged entities, but left qualifiers, including adjectives, as somewhat arbitrary judgement calls [1].

For GENETAG annotation, we chose a wide definition of a gene/protein entity, but added a constraint that requires the tagged entity to refer to a specific entity, hereafter called the "specificity constraint." The specificity constraint allows for entities like *tat dna sequence *but not *dna sequence*. No distinctions were made between genes, proteins, RNA, domains, complexes, sequences, fusion proteins, etc. A finer-grained definition is possible, for example, proteins, genes and RNA can be distinguished as separate entities using machine learning with 78–84% accuracy [3]. However, most biomedical NER systems do not make these distinctions. Also, Hatzivassiloglou et al. found that their machine learning algorithms did not perform well against a human baseline model, suggesting that either the humans were correct, and the decreased performance was due to classification difficulty, or the machine-learned programs were penalized for being more consistent than humans. Because humans agreed only about 77% of the time on protein, gene and RNA labels, the inclusion of these distinctions in a gold standard would be an additional source of significant ambiguity.

Our decision to include domains, complexes, subunits and promoters (but only if they refer to a specific gene/protein) was based on gene names in GenBank. (*Domain*: A discrete portion of a protein with its own function. The combination of domains in a single protein determines its overall function. *[Source: DOE Genome Glossary *]; *Complex*: In chemistry, the relatively stable combination of two or more compounds into a larger molecule without covalent binding; *Subunit*: A single biopolymer separated from a larger multimeric structure *[Source: Stedman's Online Medical Dictionary, 27th Edition *]; *Promoter: *a segment of DNA located at the "front" end of a gene, which provides a site where the enzymes in involved in the transcription process can bind on to a DNA molecule, and initiate transcription *[Source: Genomics Online Terms *)

For example, the name in (1) suggests that a particular subunit is considered to be a gene entity because it is associated with a GenBank sequence. Similarly, (2) shows that promoters can be considered to be gene-sequence-related entities. Our specificity constraint requires the presence of *Sf3b4 *in (1) and *transaldolase *in (2). Thus, *subunit 4 *alone in (1) and *promoter region *alone in (2) would not be true positives.

1) Sf3b4, splicing factor 3b, subunit 4

2) Mus musculus transaldolase gene, promoter region

Some exceptions to the specificity constraint were incorporated into the annotation scheme due to their appearance in GenBank:

3) bHLH transcription factor mRNA

4) Xenopus laevis similar to POU domain gene

By defining a gene based on gene names in GenBank, but requiring only a partial match, we have addressed the fact that gene names in text are often not exact matches to their official names. This is an advantage of manually annotating a corpus instead of relying on lists of official gene names for a gold standard – each entity in each context can be expertly evaluated and revised if necessary. The above examples illustrate some of the motivation behind the compilation of a list acceptable alternative gene/protein names. In (1), many systems would probably not extract the entire entity, and would be penalized. A more flexible evaluation would be possible if it were recognized that "Sf3b4" and "Sf3b4, splicing factor 3b," are acceptable alternatives to the full form. It would also allow systems to delete the organism name in (2), as well as the fact that it refers to the promoter region. The acceptable alternatives are always subject to the specificity constraint so that the important parts of gene/protein names are preserved. In addition to the specificity constraint described here, we applied semantic constraints to define gene/protein entities.

Semantic constraints are rules stating that the tagged entity must contain its true meaning in the sentence context. These constraints were geared towards multiword entities, especially ones that include numbers, letters and acronyms. For example, the name in (5) requires *rabies *because *RIG *implies that the gene mentioned in this sentence refers to the *rabies immunoglobulin*, and not just any *immunoglobulin*. In (6), the word *receptor *is necessary to differentiate *IGG receptor *from *IGG*, an important semantic distinction. In (7), the number *1 *is needed to accurately describe a specific type of *tumor necrosis factor*, although *tumor necrosis factor *alone might be adequate in a different context.

5) rabies immunoglobulin (RIG)

6) IGG receptor

7) Tumor necrosis factor 1

GENETAG consists of 20K sentences that have been run through AbGene [4] and manually annotated with gene and protein names (via a web interface) by experts in biochemistry, genetics and molecular biology. It is a heterogeneous set of sentences that contains many true positive gene names, and also many non-gene entities that are morphologically similar to gene names. There are approximately 24K instances of gene/protein names in the 20K sentences. 15K of the sentences were used in BioCreAtIvE-2004 Task 1A [5]. Previous biomedical NER systems were difficult to compare because there were few large gene-tagged corpora available. Although GENETAG was not originally intended to be widely distributed, in releasing the corpus to the larger biomedical NER community through the BioCreAtIvE Evaluation, we hoped to stimulate interest in this area and provide a means to evaluate multiple systems on unrestricted biomedical text.

GENETAG annotation guidelines were designed to define true positive gene/protein names in terms of their specificity and semantics. Each sentence in GENETAG is annotated with acceptable alternatives to the gene/protein names it contains, allowing for partial matching with semantic constraints, a more meaningful measure of the performance of an NER system than unrestricted partial matching. This paper provides some background on the corpus including 1) sentence selection, 2) definition of a gene/protein name, 3) tokenization and partial matching and 4) tagging consistency.

## Implementation

**Figure 1:**
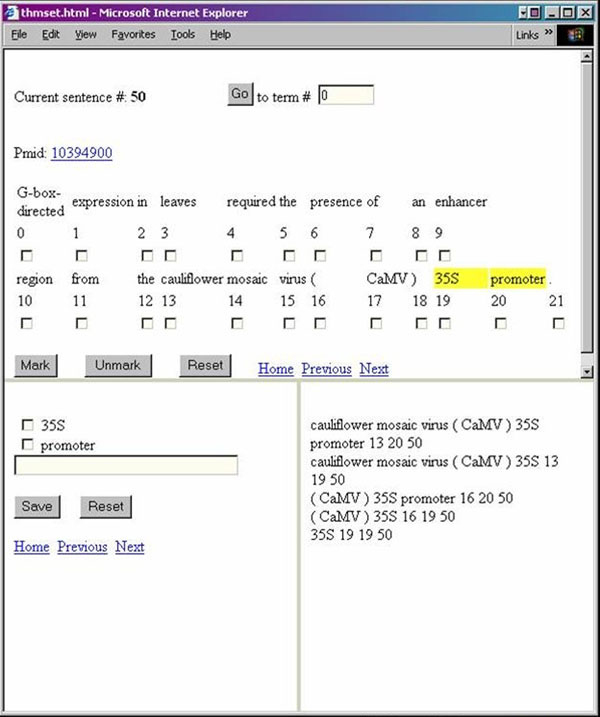
**GENETAG Annotation Method. **The annotator selects the boxes under each word in a gene/protein name and presses the "Mark" button. The resulting name is highlighted in yellow, and the marked fragments are shown in the bottom left frame. The annotator selects allowable alternatives from this list and presses "Save". Alternatives beyond the scope of the original highlighted name are input manually (along with their indices) into the text entry box. The lower right frame shows all the alternatives to the original name, along with their indices and the sentence number. A link to the abstract is provided for contextual clues.

We used Naïve Bayesian learning to predict the likelihood that a MEDLINE document contains a gene/protein name [6-8]. The classifier was trained on a set of MEDLINE documents containing known gene/protein names against the rest of MEDLINE. High-scoring documents almost always contain gene/protein names, and low-scoring documents often contain no gene/protein names. We found that we could apply our classifier to sentences as well as documents. Since we required the corpus to contain both true gene/protein names and non-gene-related entities, we randomly selected 10K high- and 10K low-scoring MEDLINE sentences as the basis for our corpus. The Bayesian classifier is implemented in C++. The 20K sentences were run through AbGene, and the resulting gene/protein tags were manually transformed by three domain experts in genetics, biochemistry and molecular biology. Annotation was done using a web interface, using check boxes under words to indicate gene/protein names for the gold standard, and manual entry of partial match alternative names and indices (see Figure 1). A flexible evaluation program was implemented in Perl.

## Results

GENETAG contains 20K sentences tagged with gene/protein names (see Table 1 for some corpus statistics). An additional file of acceptable alternatives to the tagged gene/protein names is available. The gold standard combined with the acceptable alternatives allows for flexible scoring using *meaningful *partial matching. The flexible evaluation program checks first for the gold standard version of the name, then checks for each of the available alternatives to the gold standard version of the name. Word indices ensure that specificity and semantic constraints are met for sentences that contain overlapping, repeated and/or nested names.

## Discussion

Tokenization is problematic in gene/protein name NER because punctuation cannot be globally removed to make processing straightforward. Gene and protein names often contain hyphens, parentheses, brackets, and other types of punctuation, thus using Penn Treebank style tokenization [9], where most punctuation is split from neighboring words, is not ideal. The Penn Treebank contains "subtleties" for hyphens and dashes, similarly, our original tokenization was based on rules that usually apply to gene and protein names: 1) do not split on hyphens (*Myc-2*, *c-Cbl*, *POU-domain proteins*), 2) do not split on single quotation marks (*5'-rearranged myb*, *5'-GCACGTTTT-3'*, *Marek's disease virus serotype 2 glycoprotein M*), 3) do not split on commas (*Na+,K(+)-ATPase*), and 4) do not split on parentheses and brackets (*(GST)-Lyn fusion*). AbGene applies these tokenization rules to terms that resemble gene names (phrases with commas embedded in words, matching parentheses appearing in the same word, asterisks in the middle of words, embedded semicolons, etc.), and not to other phrases (phrases with commas not embedded in words, parentheses not appearing in the same word, asterisks outside of words, semicolons outside of words, etc.), resulting in inconsistent tokenization. Some parentheses are surrounded by spaces, others are not, dependent on whether the phrase resembled a gene name or not. The corpus was later re-tokenized automatically (after annotation was completed) to be closer to the Penn Treebank style, with the exception of hyphenations and single quotation marks. Although the second tokenization is more consistent than the original tokenization, it introduces some awkward spaces into gene names, for example in (8), (9) and (10).

8) human alpha 1, 2-mannosidase

9) (IL) -1beta

10) D. melanogaster Surf-3 / rpL7a

Exact matching to a gold standard corpus is too stringent for evaluation purposes since there is not always one and only one correct answer for each entity. However, unrestricted partial matching is suboptimal because it allows unsuitable fragments with insertions and deletions to count as true positives. Canonical form matching is not possible for many organisms given current resources. In GENETAG we allowed partial matching, but the matches were subject to the specificity and semantic constraints described earlier for defining a gene/protein name. Partial matching with semantic constraints allows for acceptable gene/protein name alternatives like *p53*, *p53 genes*, *p53 protein*, but rejects fragments like *all p53 genes*, *p53 patients *and *-1beta*. Our flexible matching strategy entails that the tokenization of the gold standard corpus and test set be identical since it relies on the location of each word in the sentence. A positional approach is necessary because often one sentence contains many gene/protein names, including overlapping, repeated and/or nested names. To illustrate this problem, suppose an NER system identified the following gene in sentence fragment (11): *TGF-beta1*. It would be impossible to know which of the two instances of *TGF-beta1 *was actually extracted by the system without sentence indices. In (12), a similar ambiguity exists if an NER program were to extract the gene list: *TNF*, *Toll-like receptor*. Does *TNF *refer to *human tumor necrosis factor *or *TNF receptor 1*? Did the system extract part of *Toll-like receptor 2 *or *Toll-like receptor 4*? The exact location of gene/protein names in a sentence is essential for debugging NER programs based on contextual clues, for example, in (11) the first instance of *TGF-beta1 *is preceded by *stimulating*, and the second instance has a more generic context. The requirement that the gold standard and test sets have the same tokenization is disadvantageous. However, positional information is necessary for further natural language processing (NLP), since it affects both syntactic structure and semantic interpretation.

11) Inflammation has been inferred to play a major role in stimulating TGF-beta1 production since high concentrations of TGF-beta1 have been found in the lungs of patients with various diffuse inflammatory lung diseases.

12) RIP3-deficient cells showed normal sensitivity to a variety of apoptotic stimuli and were indistinguishable from wild-type cells in their ability to activate NF-kappa B signaling in response to the following: human tumor necrosis factor (TNF), which selectively engages mouse TNF receptor 1 ; cross-linking of the B- or T-cell antigen receptors; peptidoglycan, which activates Toll-like receptor 2 ; and lipopolysaccharide (LPS), which stimulates Toll-like receptor 4.

GENETAG was annotated manually, thus the tags assigned were judgment calls by human annotators. Annotation guidelines were established, however, many grey areas soon appeared for which no generalized rules were formulated. In particular, names containing conjunctions were difficult to tag. In (13), it seems excessive to require an NER program to recognize the entire fragment, however, *3 *alone is not a valid gene name.

13) src homology 2 and 3

Due to the specificity constraint, we were unable to formulate a syntactic rule for handling conjunctions systematically. For example, the underlined terms satisfying the specificity constraint in examples (14), (15) and (16) occur arbitrarily to the left or right of the word "or." Accepted alternatives for (14) were: *ICP34 . 5*, *ICP34 . 5 promoters*, *mutant ICP34 . 5 promoters*, *wild-type or mutant ICP34 . 5*, and *mutant ICP34 . 5*. Accepted alternatives for (15) were: *Rab1B*, *Rab1B, -5*, *Rab1B, -5, -7*, and *Rab1B, -5, -7, -8*. Accepted alternatives for (16) were: *beta, or gamma PKC*, *gamma PKC*, and *PKC*.

14) wild-type or mutant ICP34 . 5 promoters

15) Rab1B, -5, -7, -8, or -11A

16) alpha, beta, or gamma PKC

Adjoining gene/protein names also presented a challenge during annotation, since gene/protein name boundaries are not immediately obvious in these instances. It is an intricate task to assign the exact boundaries in (17) and (18), even for domain experts, and in (19) it is unclear whether *E2, RAD5 *and *UBC2 *are stand-alone synonyms or parts of a complex or fusion protein. Similarly, it is difficult even for experts to determine how many separate entities are denoted in (20). Often the sentence context, and sometimes the entire abstract context, is inadequate for the correct determination to be made, so other resources (books, websites, and full text articles) must be consulted. This time-consuming lookup step necessitates a trade-off between tagging consistency and annotation time.

17) stress-activated protein kinase-Jun N-terminal kinase

18) tumor necrosis factor (TNF) receptor-associated factor (TRAF)

19) E2 RAD5 (UBC2)

20) LAZ3/BCL6 BTB/POZ

Even in unambiguous cases, tagging inconsistencies can appear due to human error. In particular, the partial matching alternatives are sensitive to inconsistencies because the names and indices were input manually into a text box on an annotation web page (see Fig. 1).

## Conclusions

We have described the GENETAG corpus of tagged gene/protein names in MEDLINE text which was used in BioCreAtIvE Task 1A. The corpus was designed to contain both true and false positive gene/protein names in a variety of contexts. Gene/protein names are defined widely, but are subject to specificity and semantic constraints. The annotation guidelines were designed with the goal of allowing flexible matching to the gold standard, while retaining the true meaning of the tagged entities. Arbitrary partial matches not corresponding to a complete and meaningful entity fail to meet the annotation guidelines and are scored as false positives and/or false negatives. A more detailed definition of a gene/protein name, as well as additional annotation rules, could improve interannotator agreement and help solve some of the tagging inconsistencies. Subtle tokenization issues exist in the corpus, and the requirement that the gold standard and test sets have the same tokenization is disadvantageous (see discussion in [10]). However, a positional approach is necessary to disambiguate sentences which contain adjoined, repeated and/or nested gene names, and for future NLP applications. A more robust approach would use character-based rather than word-based indices to allow for a wider diversity of tokenization.

## Authors' contributions

LT outlined the annotation guidelines, annotated text, wrote the evaluation programs and drafted the manuscript. NX created the web interface for corpus annotation. LHT and WM participated in designing the annotation guidelines and annotated text. WJW conceived of the project, and participated in its design and coordination. All authors read and approved the final manuscript.

## Figures and Tables

**Table 1:** GENETAG corpus statistics The 20K sentences were split into four subsets called Train, Test, Round1 and Round2.

	**Train**	**Test**	**Round1**	**Round 2**	**Total**
**Number of Sentences**	7,500	2,500	5,000	5,000	20,000
**Number of Words**	204,195	68,043	137,586	137,977	547,801
**Number of Tagged Genes = *G***	8,935	2,987	5,949	6,125	23,996
**Total Number of Alternative Forms of Gene Names in *G***	6,583	2,158	4,275	4,505	17,531
**Number of Gene Names in G with Alternative Forms = *N***	4,675	1,522	3,057	3,186	12,440
**Average Number of Alternatives per Gene Name in *N***	1.66	1.67	1.62	1.65	1.65
